# Should Neither Sclerotherapy nor Propranolol be Used Prophylactically for Oesophageal Varices?

**DOI:** 10.1155/1992/65781

**Published:** 1992

**Authors:** A. K. Burroughs

**Affiliations:** University Department of Medicine Royal Free Hospital Pond Street London NW3 2QG UK


3. Scheele, J., Stangl, R., Altendorf-Hofman, A., Gall, F.P. *et al.* (1991) Indications of prognosis after hepatic resection for colorectal secondaries. *Surgery*, **110**, 13–23
4. Schriemer, P.A., Longnecker, D. and Mintz, P.D. (1989) The possible immunosuppression effects of perioperative blood transfusion in cancer patients. *Anesthesiology*, **68**, 422–428

### SHOULD NEITHER SCLEROTHERAPY NOR PROPRANOLOL BE USED PROPHYLACTICALLY FOR OESOPHAGEAL VARICES?

### ABSTRACT

*The PROVA Study Group. (1991) Prophylaxis of first hemorrhage from esophageal varices by sclerotherapy, propranolol or both in cirrhotic patients: A randomized multicenter trial. Hepatology;* **14**, *1016-1024*

**The objective of this randomized multicenter trial was to assess the prophylactic effect on the incidence and severity of the first variceal hemorrhage of endoscopic sclerotherapy, propranolol and the combination of the two compared with none of these treatments in patients with cirrhosis and esophageal varices. Among 819 cirrhotic patients who never had experienced variceal bleeding, esophagoscopy revealed varices in 379, of whom 286 were enrolled in the trial; 73 were allocated to sclerotherapy (paravenous polidocanol [10 mg/ml] every 1 to 2 wk until eradication), 68 to propranolol (slow-release preparation in one daily dose adjusted to provide about 25% heart rate reduction), 73 to both treatments and 72 to neither of the two treatments. The patients were observed for up to 42 months, with an average of 15 months. After variceal bleeding, patients in all groups received sclerotherapy only. The incidences of variceal bleeding ($n = 50$) were almost identical in the four groups. The relative risk (with 95% confidence limits) with sclerotherapy was 1.06 (0.61 to 1.84), and the relative risk with propranolol was 0.92 (0.53 to 1.60). The mortality rate after variceal bleeding ($n = 29$) did not differ significantly either. The mortality rate without variceal bleeding ($n = 46$) was 2.75 (1.45 to 5.22) times higher in the sclerotherapy groups than in the nonsclerotherapy groups ($p = 0.002$), whereas propranolol showed no effect, the relative risk being 1.17 (0.66 to 2.10). The total mortality rate showed no significant difference between the sclerotherapy, propranolol and control groups, but the combined therapy group had a significantly increased mortality rate.**

**This trial yielded evidence against prophylaxis of variceal hemorrhage in cirrhosis by endoscopic sclerosing injections, with or without propranolol and no support of propranolol used alone. (Hepatology 1991; 14:000-000.)**

**PAPER DISCUSSION**

KEY WORDS: Oesophageal varices, beta-blockade, sclerotherapy

This is a Danish study from the PROVA study group, which compares endoscopic sclerotherapy, propranolol, the combination of the two and a control group for the prevention and effect on severity of the first variceal haemorrhage. Previous studies of prophylactic sclerotherapy have shown discordant results, most showing no benefit or harmful effects[1]. In contrast previous propranolol and nadolol studies have shown benefit of beta-blockers[1]. Most of these trials selected patients with large varices, now known to represent those patients with an increased risk of bleeding from varices for the first time[2]. In the present study amongst 819 cirrhotics without bleeding, 379 had varices 286 of whom were randomized irrespective of variceal size. Allocation was: sclerotherapy (paravariceal 1% polidocanol) $n = 73$, propranolol (slow release, one daily dose, adjusted to provide 25% heart rate reduction) $n = 68$, both treatments $n = 73$ and none = 72. The average follow up was 15 months with a maximum of 42 months. Injections were carried out at 1 to 2 week intervals until eradication with 3 monthly endoscopy thereafter. Propranolol was given in incremental doses of 80 mg, providing the pulse remained above 50 per minute and blood-pressure above 90 mmHg. Thus sclerotherapy and propranolol were administered using presently accepted regimens.

The study is well designed and analyzed. The authors had estimated that a sample size of 440 patients would be necessary if interaction between treatments was to occur, but only 220 patients if no interaction could be demonstrated. The assumption was a 50% reduction in the rate of first bleeding would occur with prophylactic treatment (with a baseline annual rate of bleeding of 25%), with a type 1 error of 5% and type 2 of 20%. The trial was stopped following the results of the planned interim analysis at 3 years because of excess mortality in the sclerotherapy treated patients.

Variceal bleeding occurred in a similar proportion in all groups: sclerotherapy versus none - relative risk 1.06 (0.61 - 1.84, 95% confidence limits) and propranolol versus none 0.92 (0.53 - 1.6, 95% C.I.). The bleeding rate in controls was 15% comparable to that seen in the control groups of previous studies where the majority of patients had larger varices. Thus, it is unlikely that the prophylactic treatments were given to patients who were less at risk of bleeding compared to other trials. Variceal bleeding was defined as that requiring transfusion and which by endoscopy the source, was considered to be varices.

Thus bleeding not requiring transfusion was not considered a bleeding episode and neither was bleeding within 24 hours of sclerotherapy, so that there was a bias towards favouring sclerotherapy as three patients had this type of bleeding. In contrast there was only one minor bleed in the control group and one in the propranolol group. In addition there was more non-variceal bleeding in the sclerotherapy groups: 14 (12 needing transfusion) in the injected group; 9 (7 needing transfusion) in the combined group; 11 (7 needing transfusion) in the propranolol group; and 6 (4 needing transfusion) in the control group.

The mortality rate after variceal bleeding was the same, but the mortality without variceal bleeding was 2.75 times higher, 95% confidence limits (1.45-5.22) in the sclerotherapy groups compared to the non-sclerotherapy groups ($p = 0.002$), whereas propranolol had no effect. The total mortality rate was increased in the combined treatment group. This was confirmed when co-variables with an independent predictive value for mortality were assessed. This necessitated stopping the trial early. This effect of sclerotherapy was also seen in the Veterans Administration trial in the USA[3], in which the increased mortality seen with sclerotherapy, was not due to an increase in bleeding-related deaths, and in which the excess mortality disappeared once sclerotherapy was stopped. This trial was also stopped early.

In this Danish study sclerotherapy achieved eradication of varices in 66% of the group who only had injection, and in 67% of the combined treatment group. These figures are comparable to other trials and do not suggest an "aggressive" sclerotherapy programme which could be directly implicated in causing the excess deaths. Moreover, although complications of sclerotherapy were frequent these did not contribute to the mortality: oesophageal stricture ($n = 10$), ulceration ($n = 9$), mediastinitis and septicaemia ($n = 1$), aphonia ($n = 1$), recurrent pulmonary emboli ($n = 1$) and a perforation without sequelae ($n = 1$). In the propranolol group: dizziness ($n = 9$), cold extremities ($n = 8$), hypotension ($n = 7$), bradycardia ($n = 4$), orthostatic hypotension ($n = 3$), heart failure ($n = 3$), fatigue ($n = 3$), nightmares ($n = 2$), asthma ($n = 3$), acute cardiac failure ($n = 1$). These complications are similar to other beta-blocker trials. Discontinuation of propranolol occurred in only 19% of the group treated with drug alone ($n = 7$ side effects, $n = 5$ withdrawal) and 13% ($n = 3$ side effects, $n = 5$ withdrawal) of the combined group again similar to previous trials. These two aspects of beta-blockade do not suggest that the drug treatment groups had a more favourable response than in other trials, thus potentially accentuating the differences between beta-blockers and sclerotherapy. Moreover, these aspects do not explain the lack of therapeutic efficacy of propranolol seen in this trial.

The authors comment that their results with propranolol are compatible with the range of effect evidenced from an updated meta-analysis of beta-blocker trials[4]. Thus the different result may simply be due to random sampling variation. The overviews of sclerotherapy and beta-blockers trials[1,4] show that beta-blockers do significantly reduce the risk of first bleeding from varices, with a borderline effect on survival. No beta-blocker trial has a significantly increased mortality. In contrast the sclerotherapy trials show statistical heterogeneity in the meta-analysis. Some of the largest trials show an increased mortality in the sclerotherapy groups. This meta-analytical data, re-enforced by this trial, indicate that sclerotherapy should not be used for the primary prophylaxis of variceal bleeding and that beta-blockers do no harm and are likely to be beneficial. They are the treatment of choice. The reasons for the excess mortality in the sclerotherapy treated patients are not clear, but this raises the issue of whether it may occur in patients whose varices are injected for the prevention of variceal re-bleeding.

Dr A K Burroughs
University Department of Medicine
Royal Free Hospital
Pond Street
London NW3 2QG, UK

### REFERENCES

1. Pagliaro, I., Burroughs, A.K., Sorensen, T.I.A., Lebrec, D., Morabito, A., D'Amico, G. and Tine, F. (1989) Therapeutic controversies and randomised controlled trials: prevention of bleeding and re-bleeding in cirrhosis. *Gastroenterology International* 2, 71–84
2. North Italian Endoscopic Club for the Study and Treatment of Esophageal Varices (1988) Prediction of the first variceal haemorrhage in patients with cirrhosis of the liver and esophageal varices. *N.Engl.J.Med.*, **319**, 983–989
3. The Veterans Affairs Co-operative Variceal Sclerotherapy Group (1991) Prophylactic sclerotherapy for oesophageal varices in men with alcoholic liver disease. *N.Engl.J.Med.*, **324**, 1779–1784
4. Pagliaro, L., Burroughs, A.K., Sorensen, T.I.A., Lebrec, D., Morabito, A., D'Amico, G. and Tine, F. (1990) Beta-blockers for preventing variceal bleeding. *Lancet*, **336**, 1001–1002

### EFFECT OF ALCOHOL CONSUMPTION ON VARICEAL REBLEEDING AND MORTALITY

### ABSTRACT

*McCormick, P.A., Morgan, M.Y., Phillips, A., Yin, T.P., McIntyre, N. and Burroughs, A.K. (1992) The effects of alcohol use on rebleeding and mortality in patients with alcoholic cirrhosis following variceal haemorrhage. Journal of Hepatology;* 14, *99–103.*

**The effect of continued alcohol intake on prognosis in alcoholic cirrhotics who have already bled from varices is controversial. To investigate the effect of alcohol intake on prognosis we studied 189 consecutive alcoholic cirrhotics admitted, for the first time, to the Royal Free Hospital with variceal bleeding. Sixty-six died within 30 days of admission and 23 were excluded from the study for other reasons. Of the 100 remaining 15 remained 'probably abstinent' over long-term follow-up, 29 drank occasionally and 56 continued to misuse/abuse alcohol. The percentage survival probability at 2 years was 66% in the probable abstainers, 68% in the occasional drinkers and 63% in the alcohol abuse/misuse group. There were no significant differences in either mortality or rebleeding rates between the three groups. A rebleeding index (designed to take account of the number of rebleeds per patient and the total length of follow-up) also failed to show any significant difference between the three groups. The Cox proportional hazard model was used to study the effect of the following factors on rebleeding and mortality; age, sex, alcohol use, Pugh's score, acute treatment received for initial variceal bleed and long-term treatment received for prevention of recurrent variceal haemorrhage. Pugh's score was significantly related to risk of death during follow-up ($p = 0.0122$), but none of the others factors was significantly related to risk of rebleeding or mortality. Using conventional methods to determine alcohol use we were unable to demonstrate significant effects of alcohol intake on rebleeding or mortality in alcoholic cirrhotics who had bled from oesophageal varices.**

